# Supplementary material for: AIRE is induced in oral squamous cell carcinoma and promotes cancer gene expression
Source: PLoS One. 2020 Feb 3;15(2):e0222689. doi: 10.1371/journal.pone.0222689 (PMC6996854; doi:10.1371/journal.pone.0222689)
Supplement: S1 Table — (PDF) [file pone.0222689.s001.pdf]

S1 Table

Primers for RT-PCR

AIRE: GAGACGCTTCATCTGAAGGAA / CTGACTCAAACACCTGCTGGAT
GAPDH: GCACCGTCAAGGCTGAGAAC / ATGGTGGTGAAGACGCCAGT
CXCL10: AGGAACCTCCAGTCTCAGCA / ATTTTGCTCCCCTCTGGTTT
CXCL11: AGAGGACGCTGTCTTTGCAT / TGCTTCGATTTGGGGTTTAG
MMP9: GATGCGTGGAGAGTCGAAAT / CACCAAACTGGATGACGATG

Primers for ChIP

STAT1: AAGGCTGCCCTGATATGTTG / GCCTTCCTAGCATCAGGTTG
ICAM1: CGGTGTAGACCGTGATTCAA / GCTGCAGTTATTTCCGGACT
CXCL10: ACCTCCTGTTTCTGGGGACT / TAGGGAGGGAAAATGGCTTT
MMP9: CTGGAGGCTTTCAGACCAAG / CAGTCTCCACTGCCAAGTCA
GAPDH: GGGTCTTTGCAGTCGTATGG / ACCTCCTGTTTCTGGGGACT
