# Supplementary figures and images for: AIRE is induced in oral squamous cell carcinoma and promotes cancer gene expression
Source: PLoS One. 2020 Feb 3;15(2):e0222689. doi: 10.1371/journal.pone.0222689 (PMC6996854; doi:10.1371/journal.pone.0222689)

S1 Fig

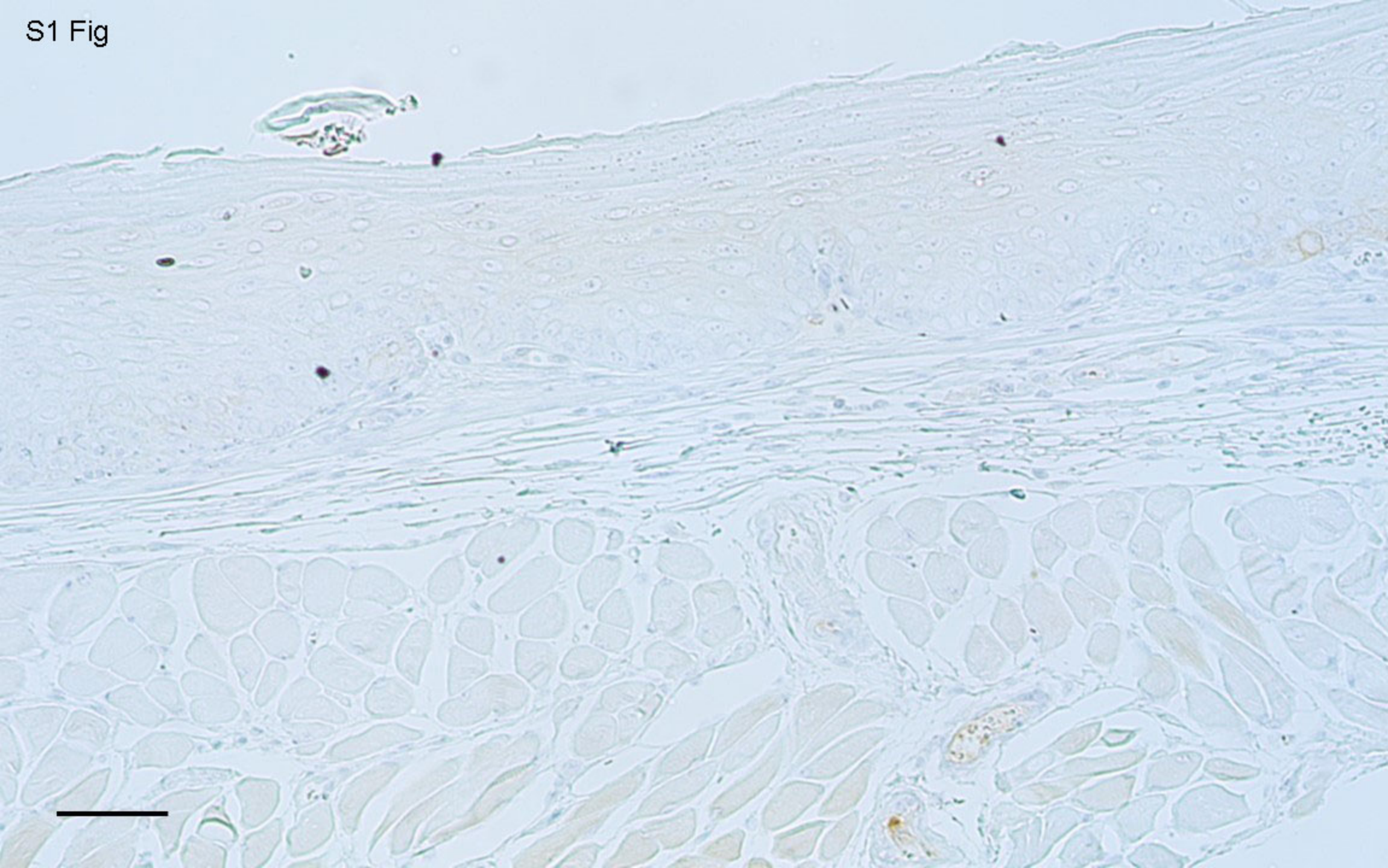

Supplement: S1 Fig — Scale bar: 100 μm. (PDF) [file pone.0222689.s002.pdf]

S2 Fig

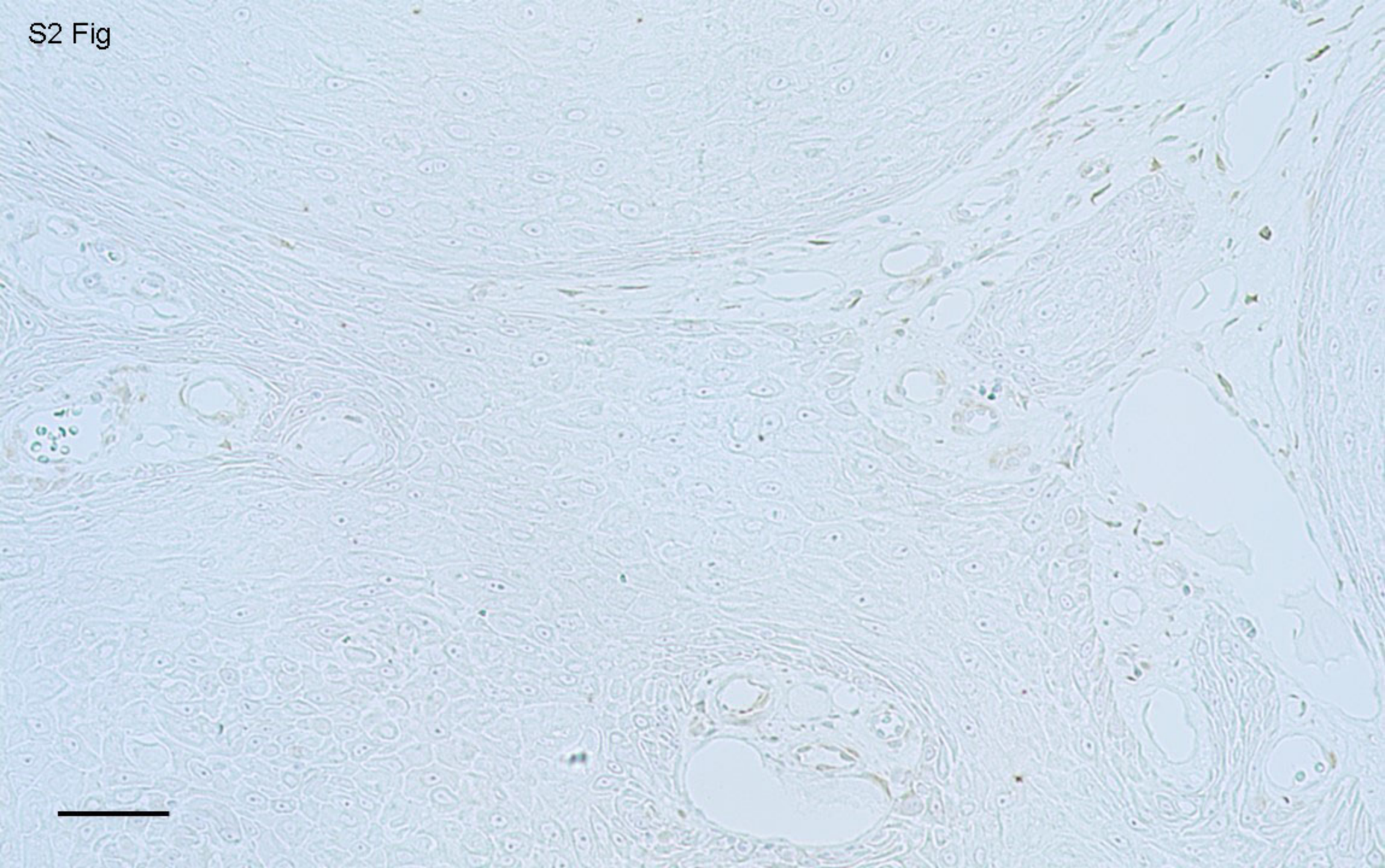

Supplement: S2 Fig — Scale bar: 100 μm. (PDF) [file pone.0222689.s003.pdf]

S3 Fig

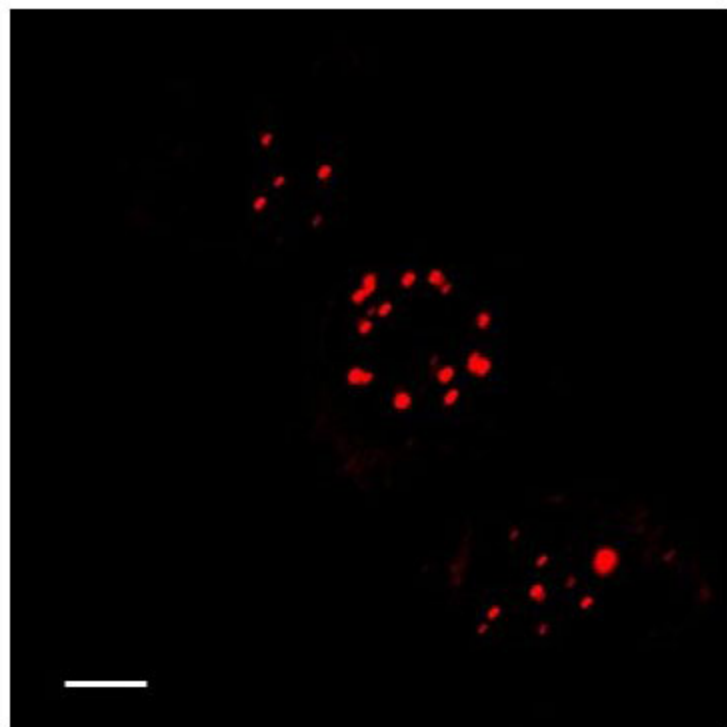

SP100

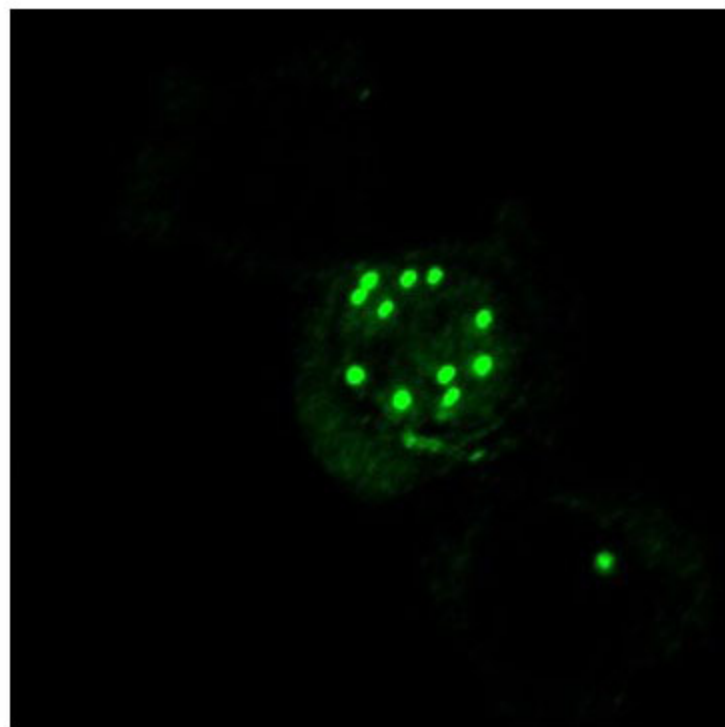

AIRE

Supplement: S3 Fig — There is a transfected cell in the center, in which AIRE colocalizes with endogenous Sp100. Note the Sp100 expression in the surrounding non-transfected cells. Scale bar: 5 μm. (PDF) [file pone.0222689.s004.pdf]

S4 Fig

Ca9-22 HSC3 HSC4 HSC5 H01N1 SAS BHY

STAT1

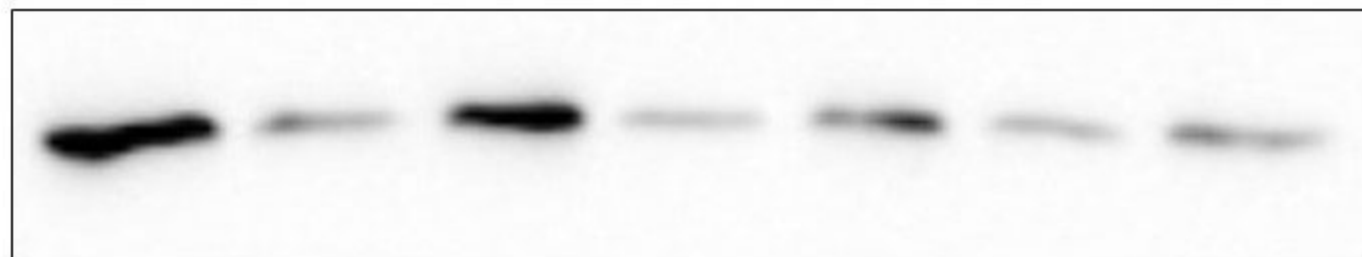

pSTAT1

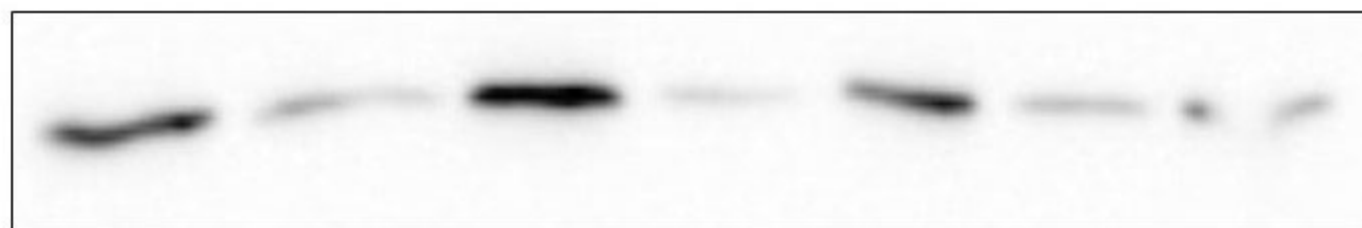

ICAM1

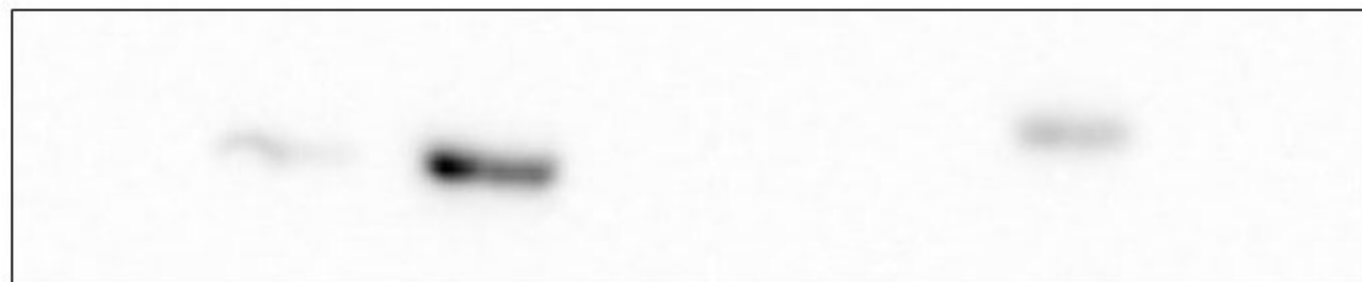

ETS1

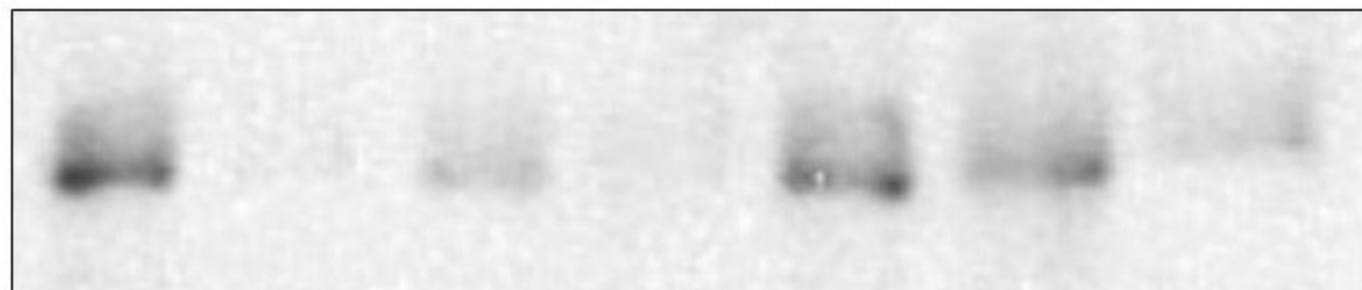

GAPDH

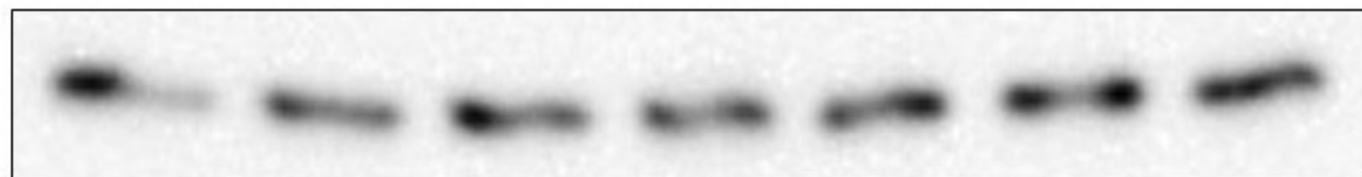

Supplement: S4 Fig — Western blot analysis. GAPDH was used as a loading control. (PDF) [file pone.0222689.s005.pdf]
