# Supplementary material for: AIRE is induced in oral squamous cell carcinoma and promotes cancer gene expression
Source: PLoS One. 2020 Feb 3;15(2):e0222689. doi: 10.1371/journal.pone.0222689 (PMC6996854; doi:10.1371/journal.pone.0222689)
Supplement: S1 File — (PDF) [file pone.0222689.s006.pdf]

1. NE 2. Ca9-22 3. HSC3 4. HSC4 5. HSC5 6. HO1N1
7. SAS 8. BHY

Fig_2B

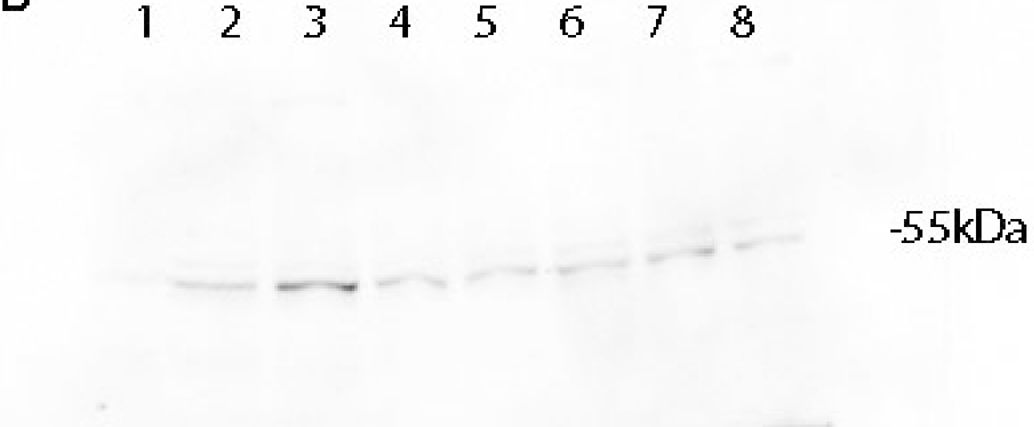

1. NE 2. Ca9-22 3. HSC3 4. HSC4 5. HSC5 6. HO1N1 7. SAS 8. BHY

Fig_2B

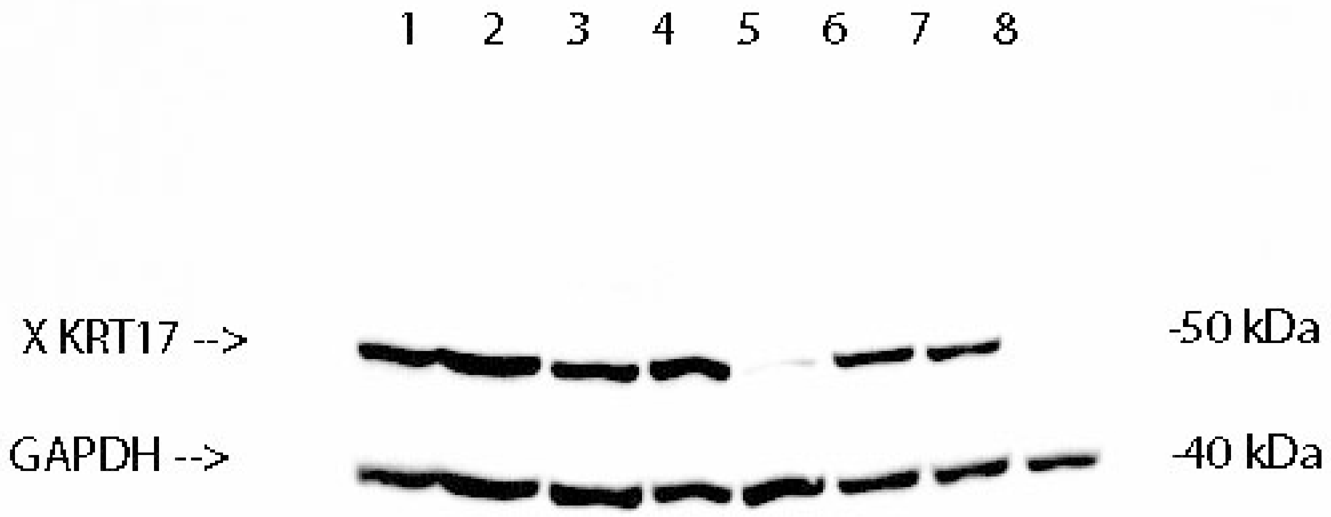

Fig_3B

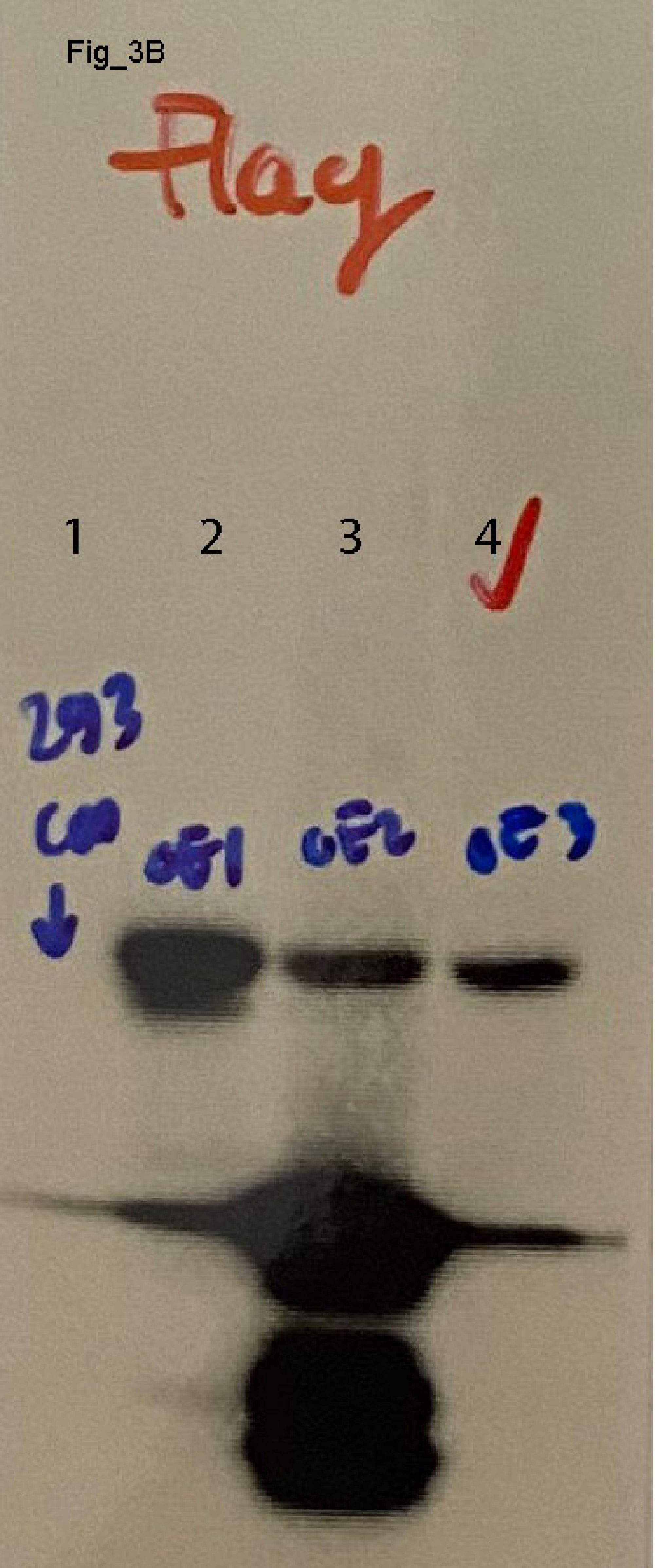

1. 293A

293/AIRE+
2. C1
3. C2
4. C3

-55 kDa

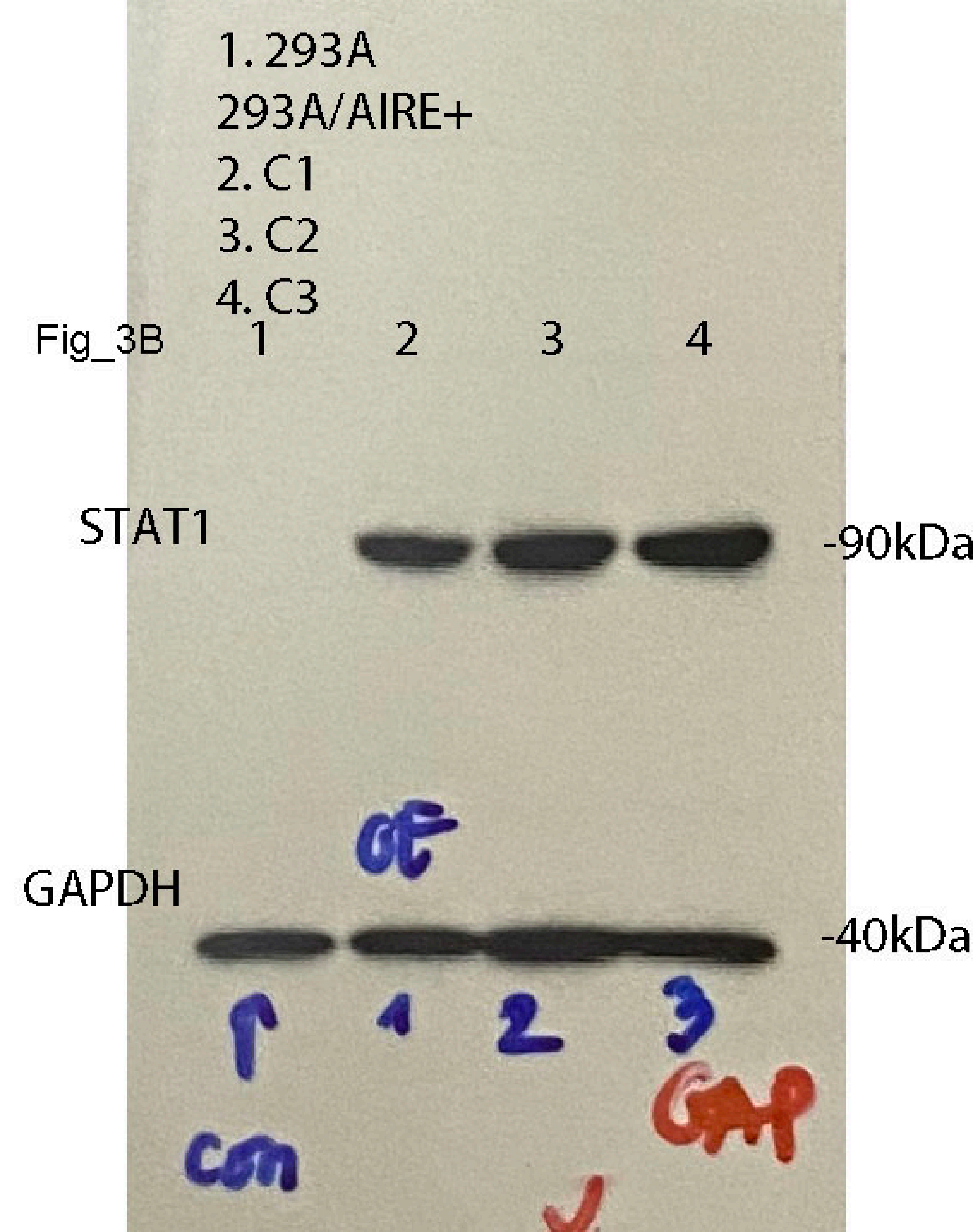
1. 293A
293A/AIRE+
2. C1
3. C2
4. C3
Fig_3B
1
2
3
4
STAT1
-90kDa
GAPDH
-40kDa
con
GAP

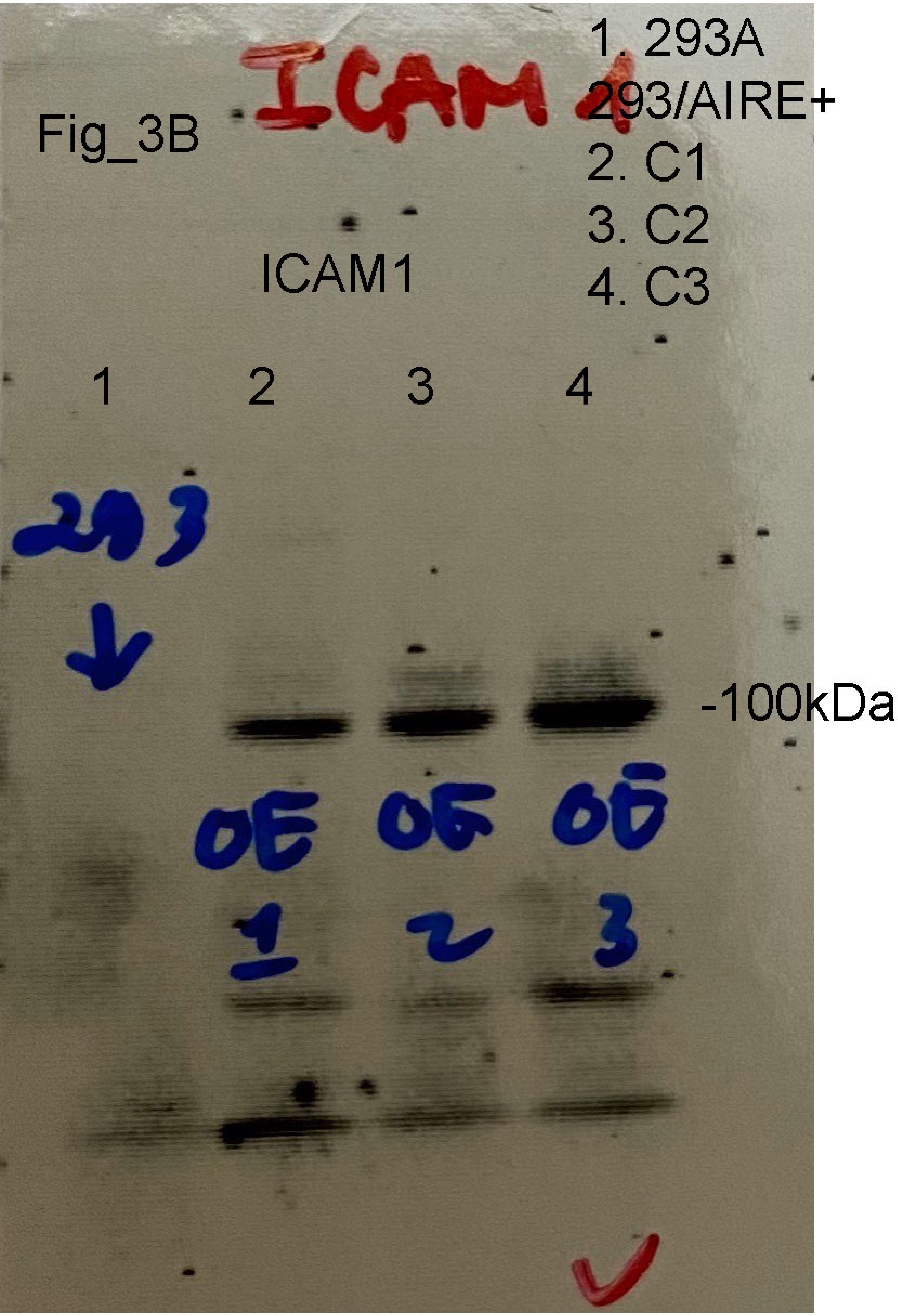
Fig_3B
ICAM1
1. 293A
293/AIRE+
2. C1
3. C2
4. C3
1
2
3
4
-100kDa

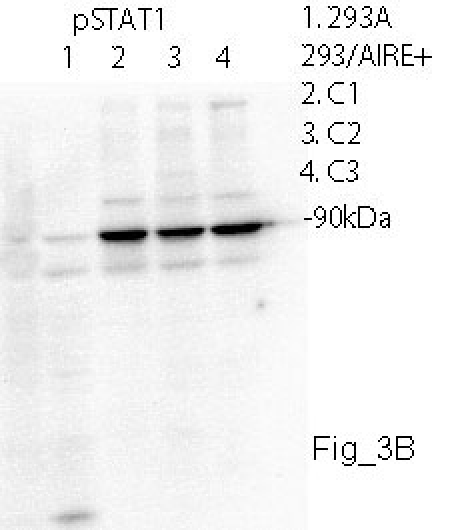
pSTAT1
1 2 3 4
1. 293A
293/AIRE+
2. C1
3. C2
4. C3
-90kDa
Fig_3B

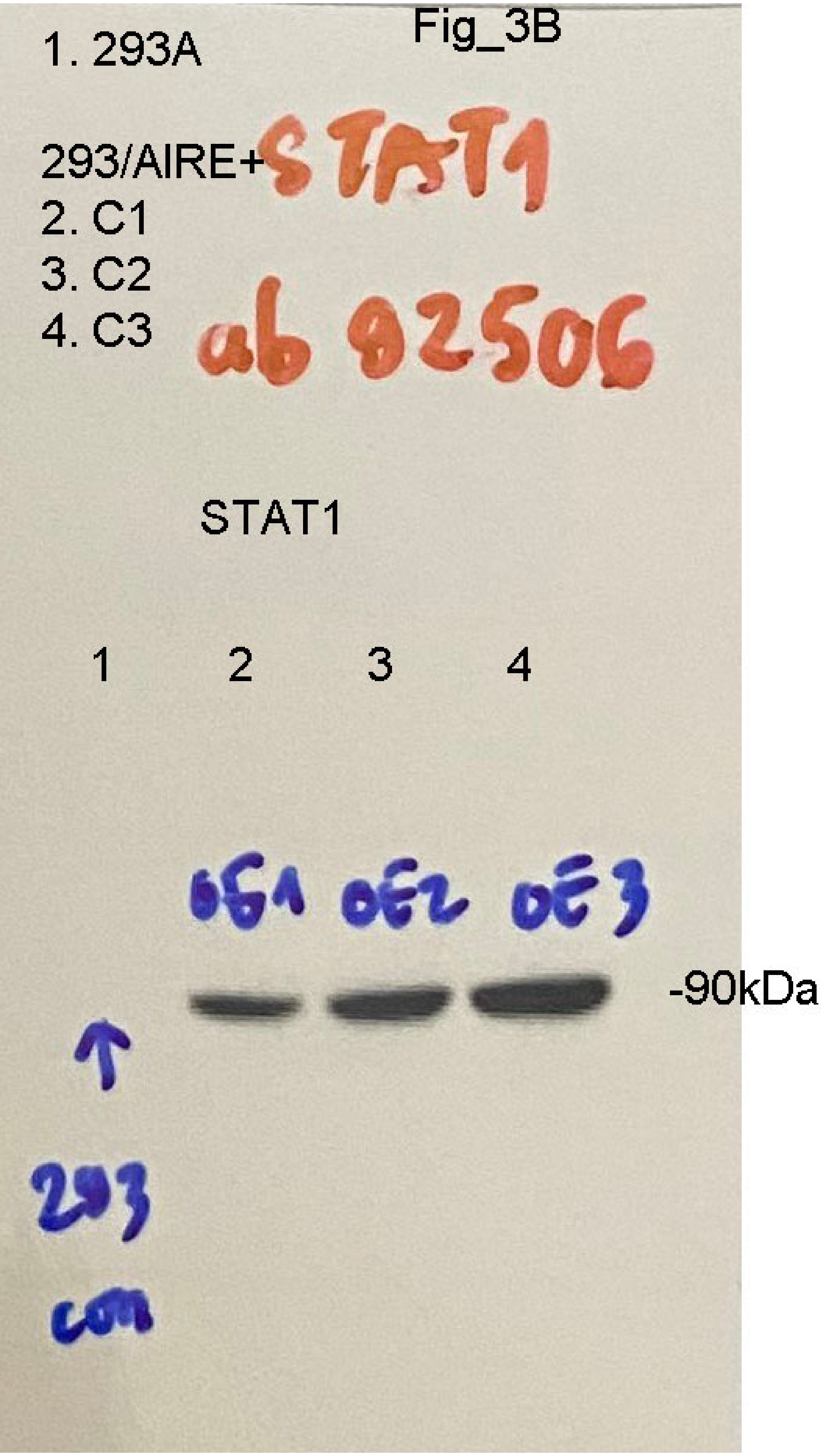

Fig_3B
1. 293A
293/AIRE+
2. C1
3. C2
4. C3
STAT1
ab 92506
STAT1
1
2
3
4
OE1
OE2
OE3
-90kDa
293
con

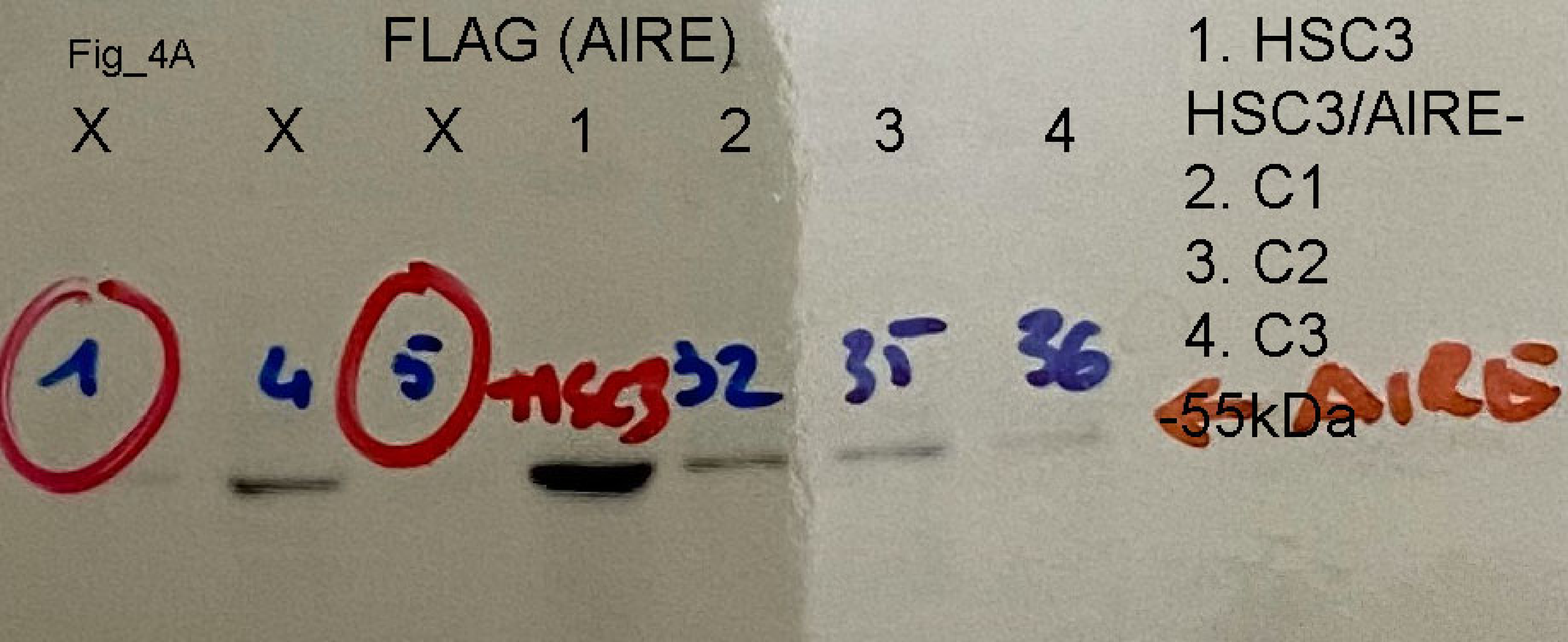
Fig_4A
FLAG (AIRE)
X X X 1 2 3 4
1. HSC3
HSC3/AIRE-
2. C1
3. C2
4. C3
-55kDa

Fig_4A

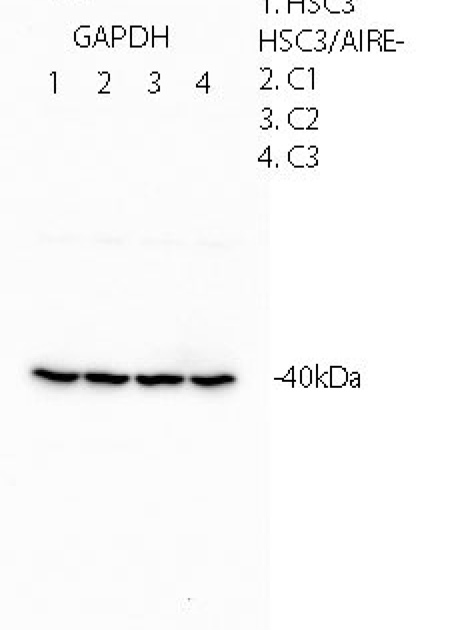

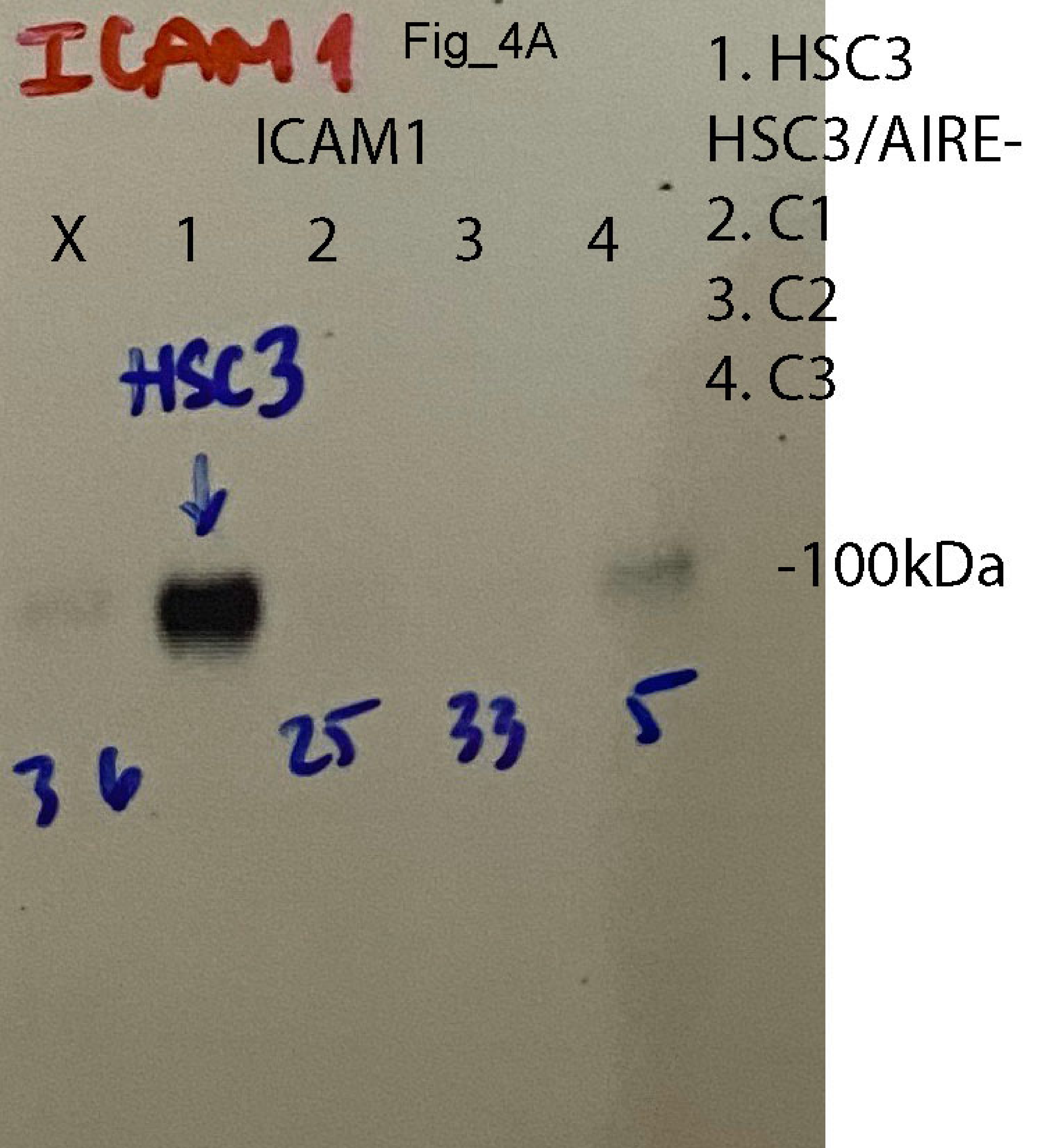
ICAM1
Fig_4A
1. HSC3
HSC3/AIRE-
2. C1
3. C2
4. C3
ICAM1
X
1
2
3
4
HSC3
-100kDa
3 6
25
33
5

Fig_4A

1. HSC3
HSC3/AIRE-
2. C1
3. C2
4. C3

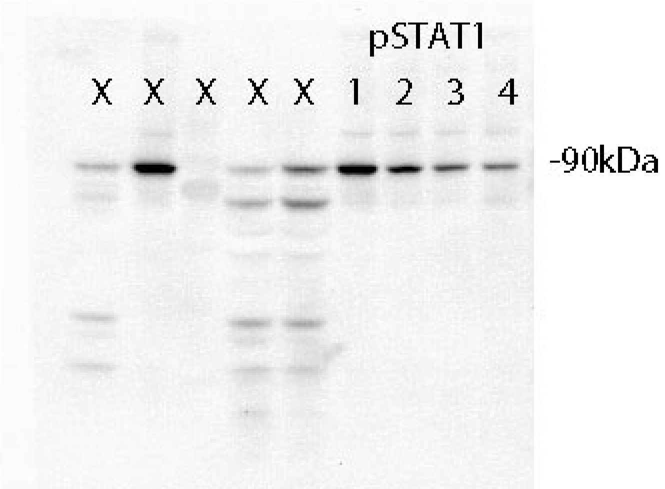

Fig_4A

1. HSC3
HSC3/AIRE-
2. C1
3. C2
4. C3

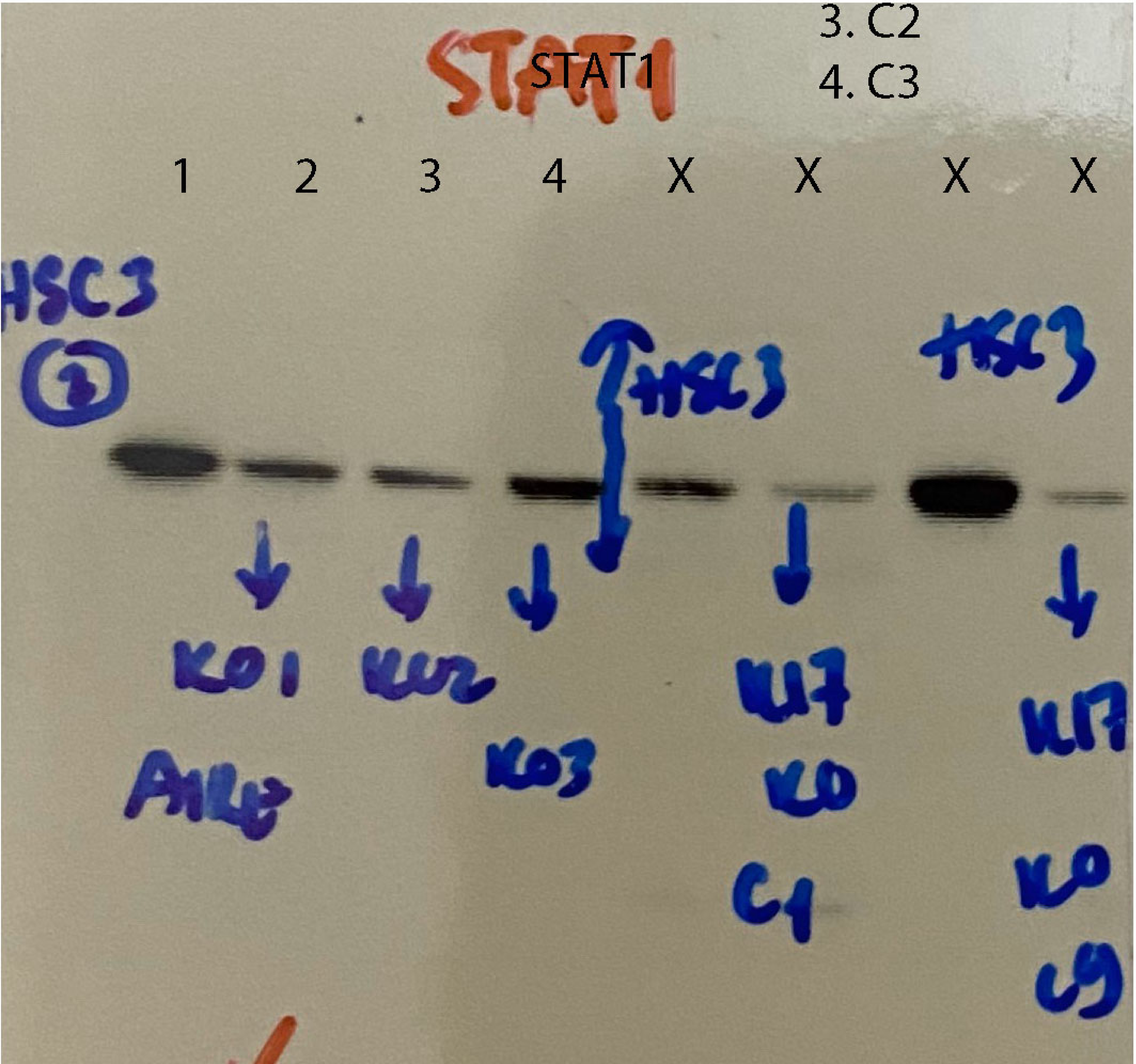

90kDa

Fig6: AIRE

1 Mock 2 AIRE 3 Ets1 4 AIRE+Ets1

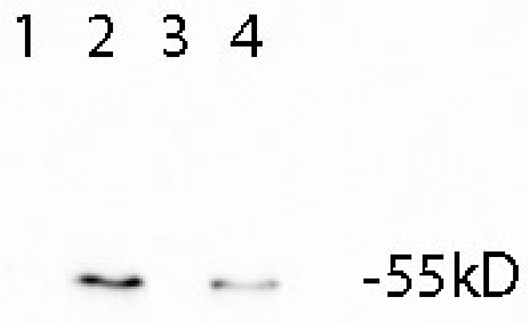

FLAG (AIRE)

Fig6: ETS1
1 Mock 2 AIRE
3 Ets1 4 AIRE+Ets1

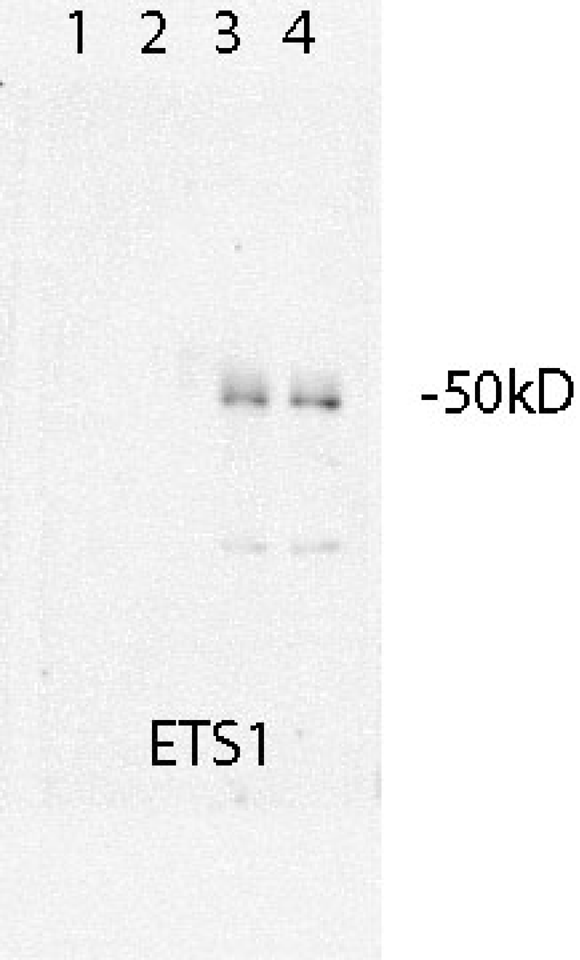

Fig6A: GAPDH

1 Mock 2 AIRE 3 Ets1 4 AIRE+Ets1

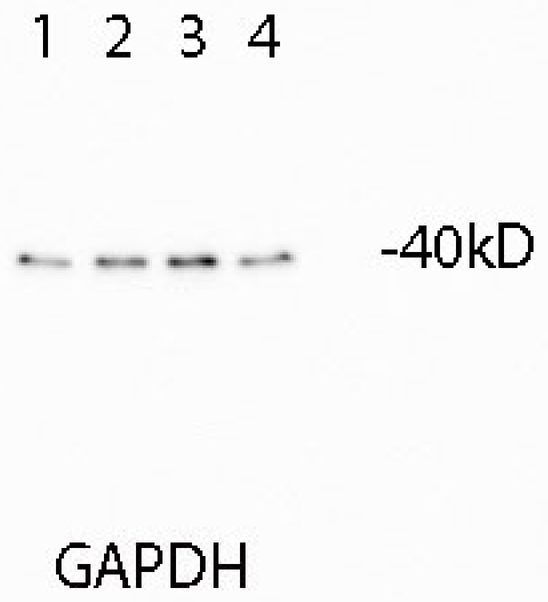

Fig6A: ICAM1

1 Mock 2 AIRE 3 Ets1 4 AIRE+Ets1

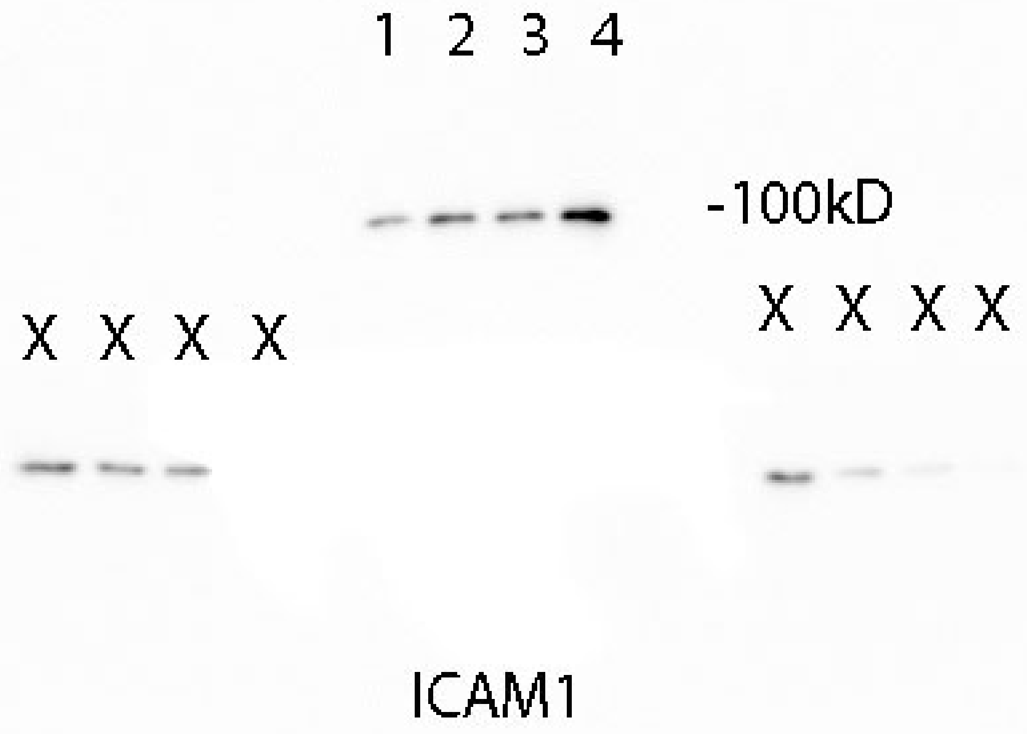

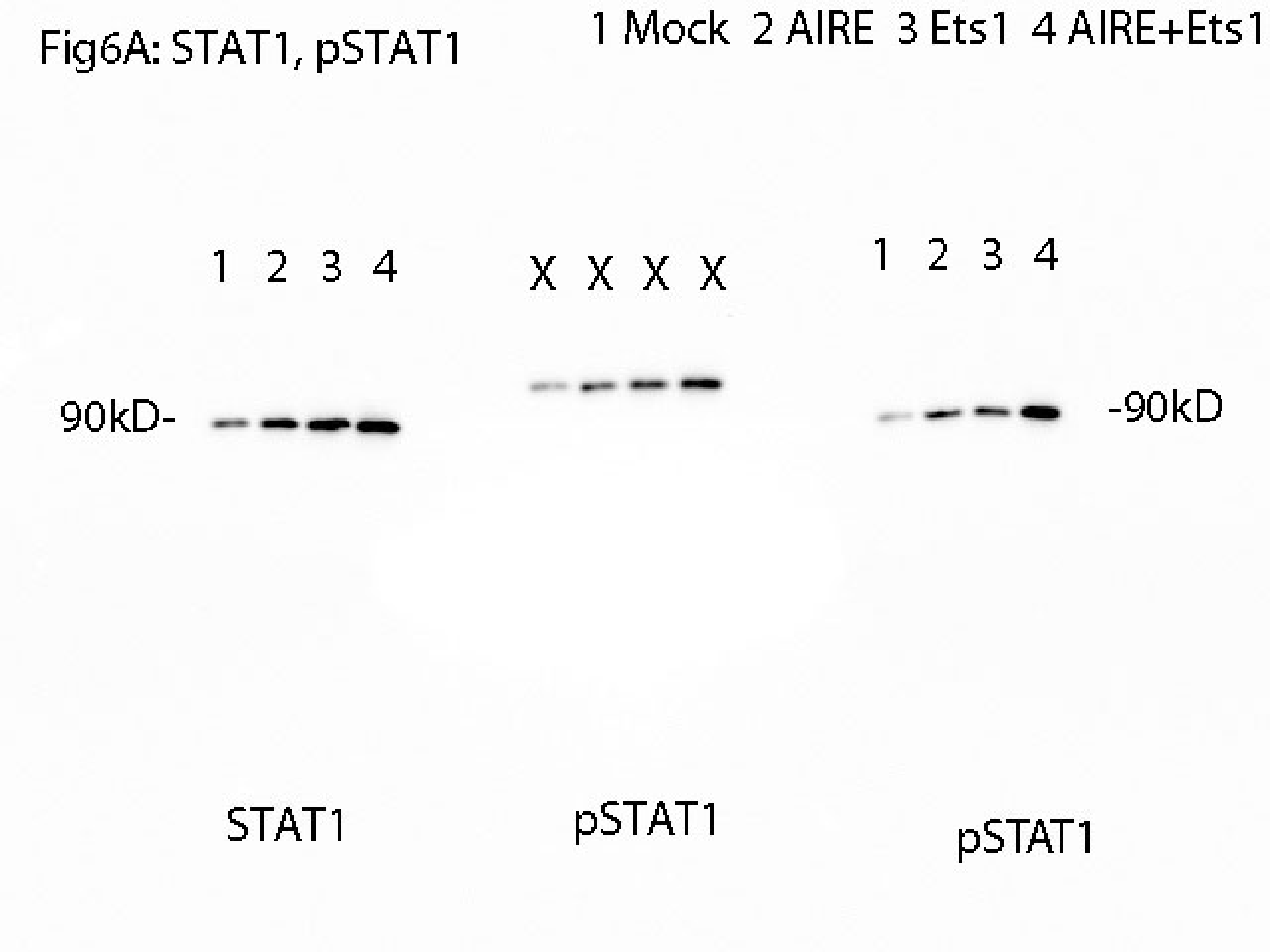
Fig6A: STAT1, pSTAT1
1 Mock 2 AIRE 3 Ets1 4 AIRE+Ets1
1 2 3 4
X X X X
1 2 3 4
90kD-
-90kD
STAT1
pSTAT1
pSTAT1

1 AIRE | IP: ETS1
2 Ets1 | IP: ETS1
3 AIRE+Ets1 | IP: ETS1
4 AIRE | 1/10 input
5 Ets1 | 1/10 input
6 AIRE+Ets1 | 1/10 input

Fig_6D

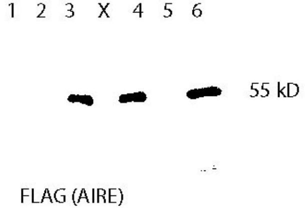

1 AIRE | IP: ETS1
2 Ets1 | IP: ETS1
3 AIRE+Ets1 | IP: ETS1
4 AIRE | 1/10 input
5 Ets1 | 1/10 input
6 AIRE+Ets1 | 1/10 input

X size marker

Fig_6D

1 2 3 X 4 5 6

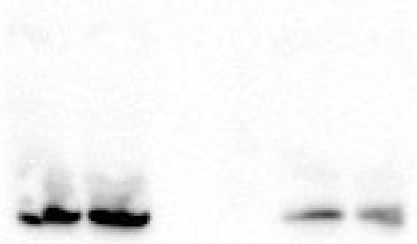

50 kD

ETS1

1 Ca9-22 2 HSC3 3 HSC4
4 HSC5 5 HO1N1 6 SAS
7 BHY

S4_Fig: ETS1

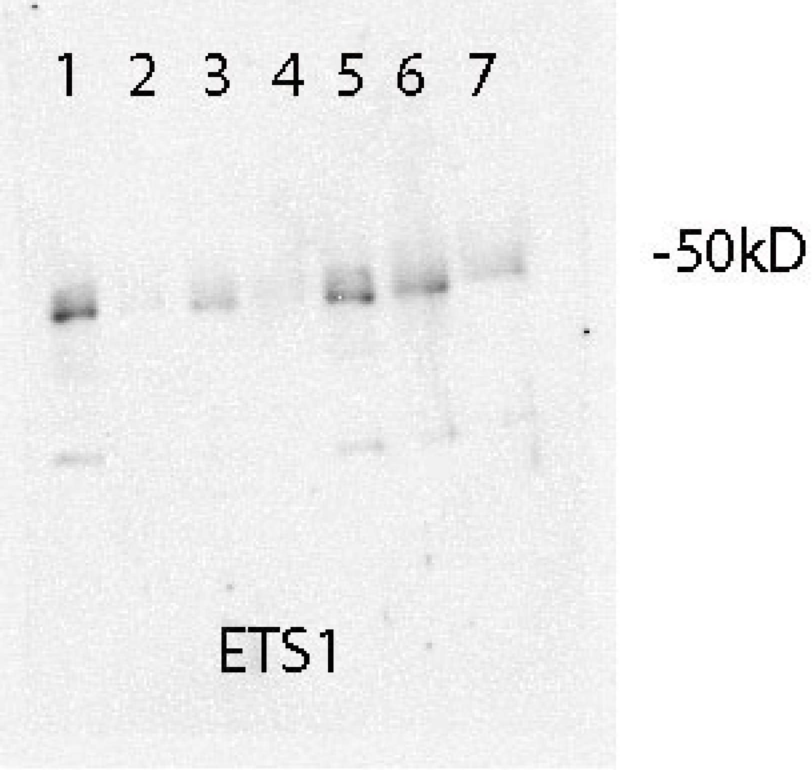

S4_Fig: GAPDH

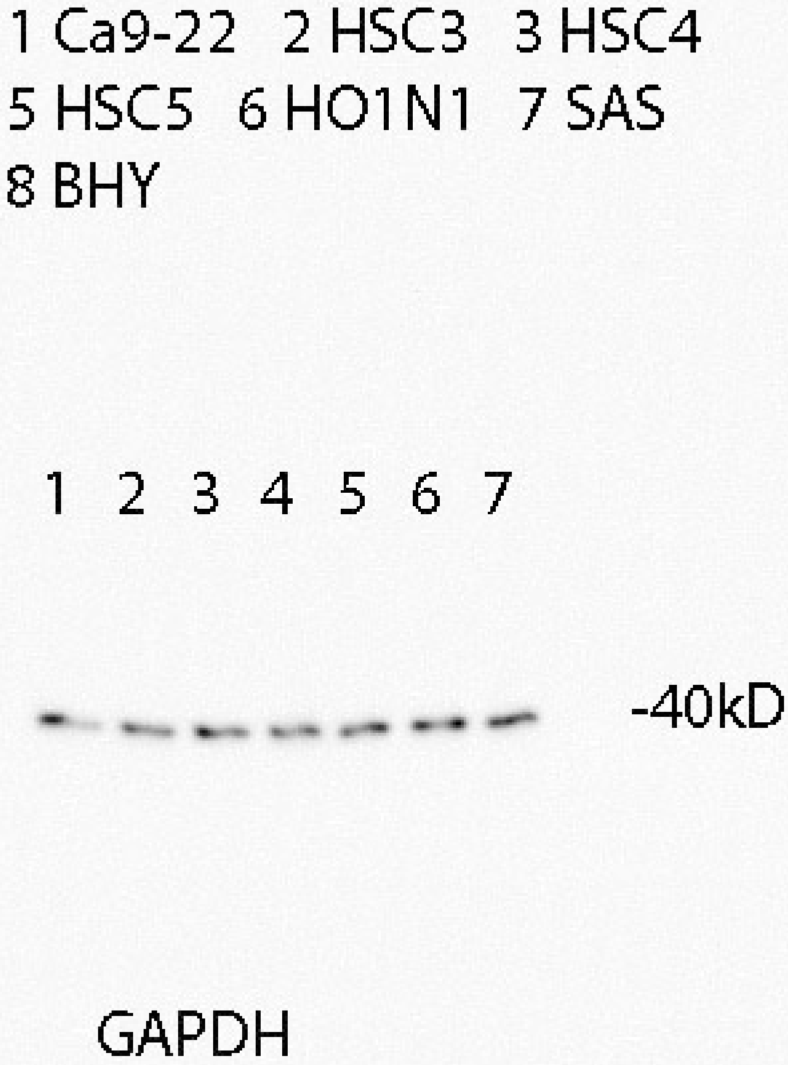
1 Ca9-22 2 HSC3 3 HSC4
5 HSC5 6 HO1N1 7 SAS
8 BHY
1 2 3 4 5 6 7
-40kD
GAPDH

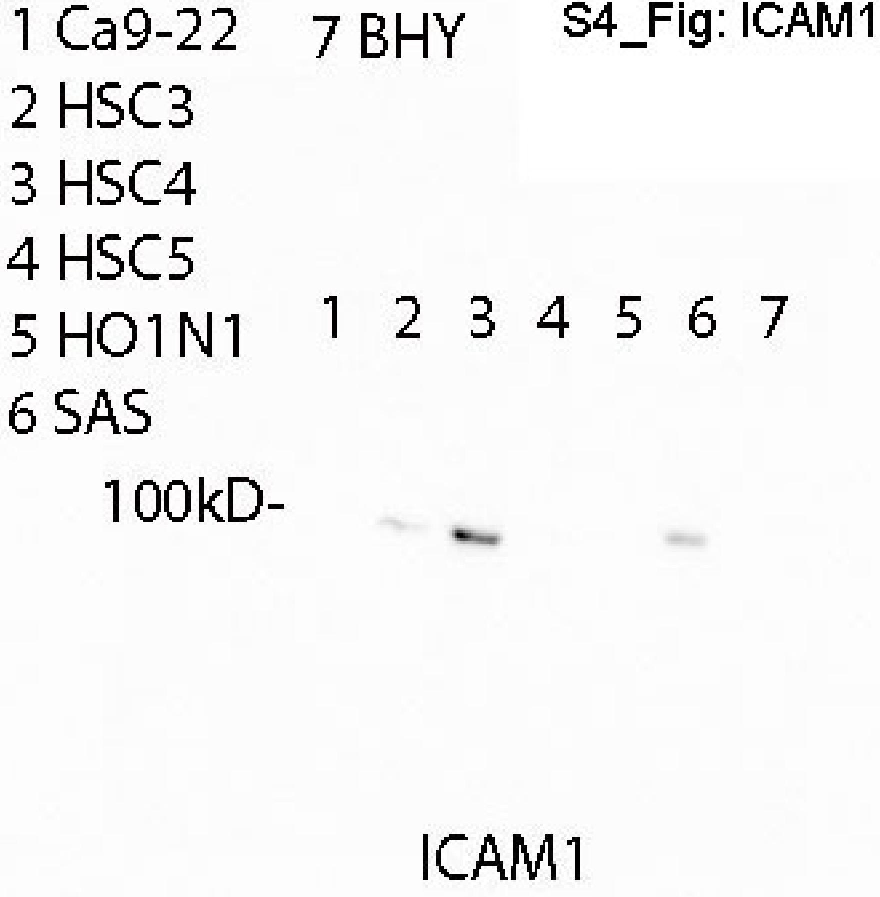
S4_Fig: ICAM1
1 Ca9-22
2 HSC3
3 HSC4
4 HSC5
5 HO1N1
6 SAS
7 BHY
1 2 3 4 5 6 7
100kD-
ICAM1

S4_Fig: pSTAT1

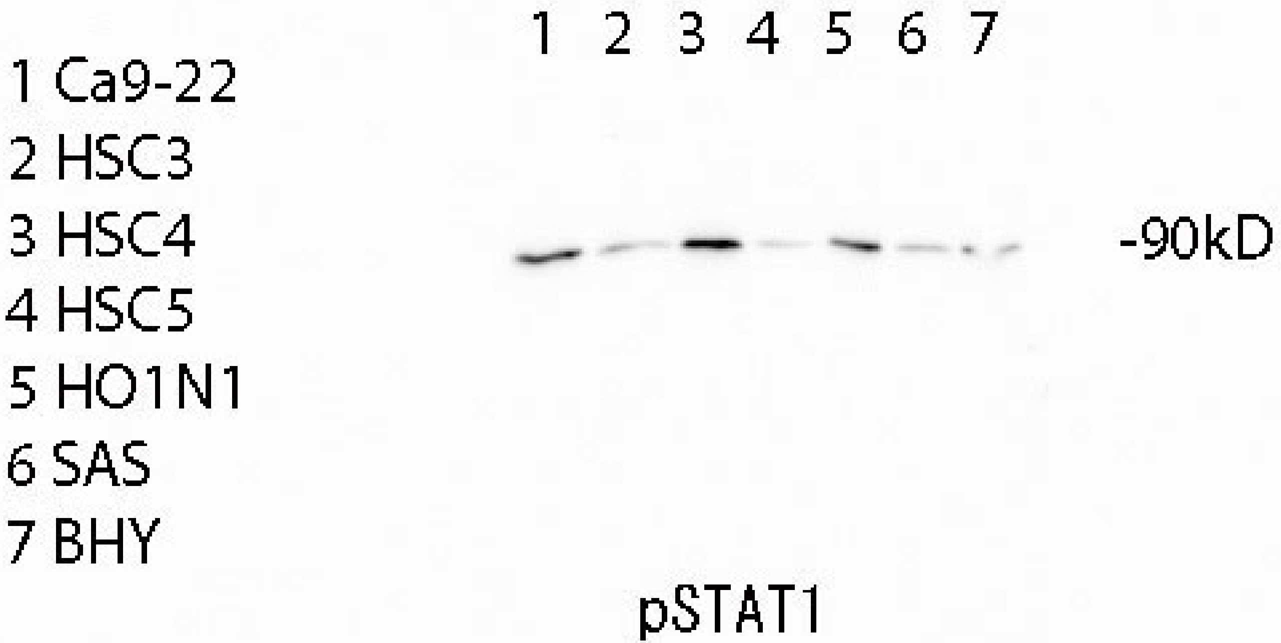

S4_Fig: STAT1

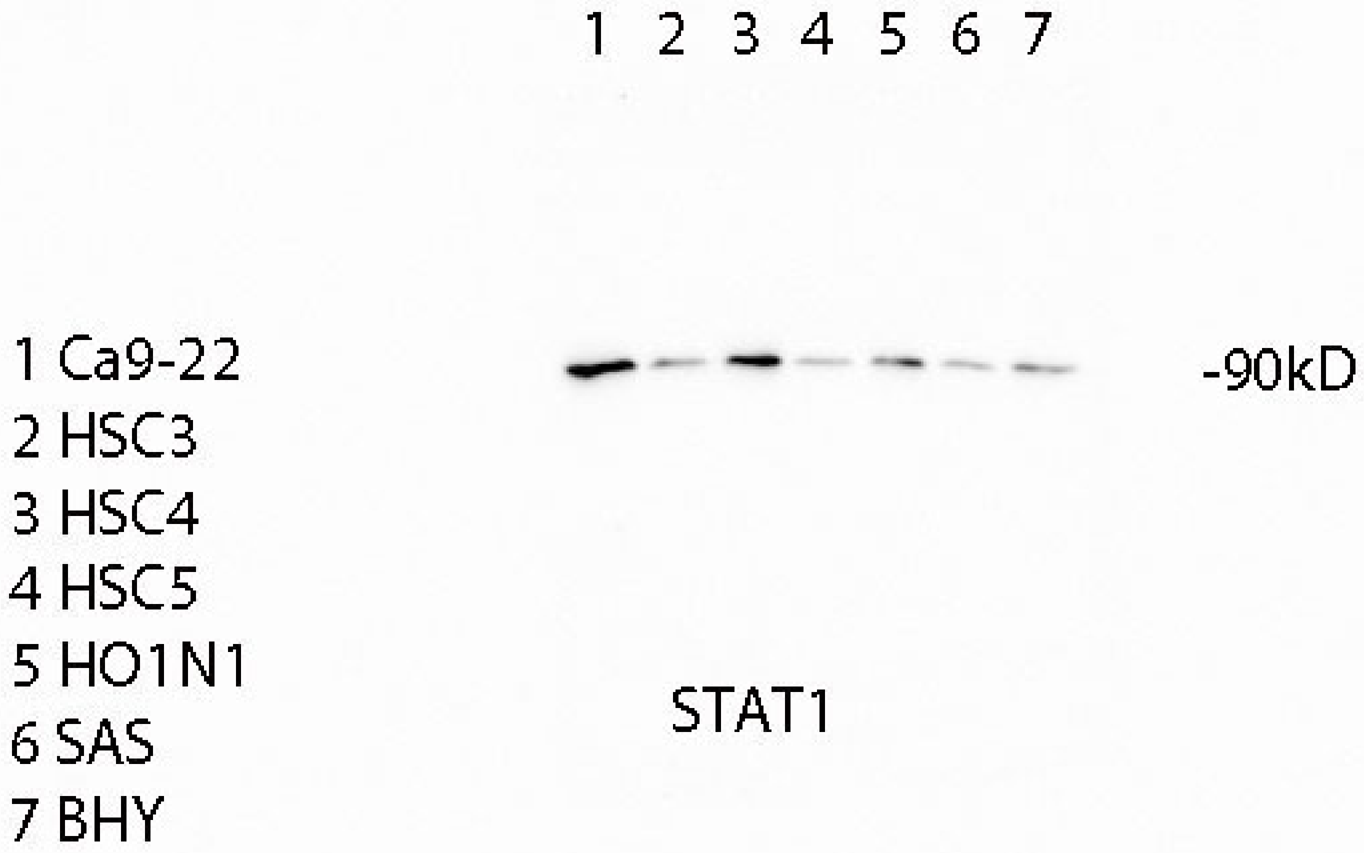
1 2 3 4 5 6 7
1 Ca9-22
2 HSC3
3 HSC4
4 HSC5
5 HO1N1
6 SAS
7 BHY
-90kD
STAT1
