# Supplementary material for: AIRE is induced in oral squamous cell carcinoma and promotes cancer gene expression
Source: PLoS One. 2020 Feb 3;15(2):e0222689. doi: 10.1371/journal.pone.0222689 (PMC6996854; doi:10.1371/journal.pone.0222689)
Supplement: Attachment — Submitted filename: Sakamoto revision.docx [file pone.0222689.s007.docx]

The manuscript *AIRE is induced in oral squamous carcinoma and promotes cancer gene expression* by Sakamoto K et al. addresses the role of ETS1, KRT17 and AIRE transcriptional factor in extrathymic tissues under pathological conditions.

The work is not particularly novel, however it extends previous published studies by investigating this pathway in oral carcinomas.

Data from Hobbs RP et al. Nat. Genetics 2015 doi: 10.1038/ng.3355 demonstrated that AIRE induction and nuclear localization strongly depends upon functional interaction between Krt17 and hnRNPK. In this work, instead, the authors describe induction of Krt17 by AIRE thus suggesting a direct role of AIRE transcriptional factor in Krt17 induction. Thus, a critical point should be to understand the relevance of Krt17 in AIRE-induced transcriptional pathway or the potential existence of a regulatory feedback loop between them, a relevant point that has not been addressed by the authors.

The work is potentially interesting however I have several concerns especially about the way the data have been done and presented.

**Major points.**

1.The author should test if Krt17 knock-out or depletion plays any effects on AIRE-dependent gene regulation. This should easily clarify the role of Krt17 in this pathway.

2.Experiments in Fig. 2A and B should be quantitated and statically evaluation of the data should be provided.

In Fig. 3A, a panel showing mock transfected cells is missing. The experiment in Fig. 3C is not quantitative and should be repeated by quantitative PCR with appropriate internal controls.

In Fig. 3D the authors should explain why expression of Krt17 in clone C1 is so low given that AIRE is abundantly expressed compared to the other clones. Again, this experiment is not quantitative and statistics is not present.

In Fig. 4B, STAT1 levels appear to be comparable despite the different level of AIRE in C1, C2 and C3 clones. The WB presented in Fig. 4B is not quantitated.

The WB in Fig. 7 should be quantitated, the AIRE panel in Fig. 7B

In general Figures are of low quality and the article does not meets the scientific criteria to be accepted for publication in PlosONE.
